# Supplementary material for: Observation of entanglement transition of pseudo-random mixed states
Source: Nat Commun. 2023 Apr 8;14:1971. doi: 10.1038/s41467-023-37511-y (PMC10082798; doi:10.1038/s41467-023-37511-y)
Supplement: Supplementary file 1 — Supplementary Information [file 41467_2023_37511_MOESM1_ESM.pdf]

Supplementary Information for "Observation of entanglement negativity transition of pseudo-random mixed states"

Tong Liu,[1,2] Shang Liu,[3] Hekang Li,[1] Hao Li,[1] Kaixuan Huang,[1,4] Zhongcheng Xiang,[1,2,4,5,6,7] Xiaohui Song,[1,2,4,5,6,7] Kai Xu,[1,2,4,5,6,7,*] Dongning Zheng,[1,2,4,5,6,7,†] and Heng Fan[1,2,4,5,6,7,‡]

[1]*Institute of Physics, Chinese Academy of Sciences, Beijing 100190, China*
[2]*School of Physical Sciences, University of Chinese Academy of Sciences, Beijing 100190, China*
[3]*Kavli Institute for Theoretical Physics, University of California, Santa Barbara, California 93106, USA*
[4]*Beijing Academy of Quantum Information Sciences, Beijing 100193, China*
[5]*Hefei National Laboratory, Hefei 230088, China*
[6]*CAS Center of Excellence for Topological Quantum Computation, University of Chinese Academy of Sciences, Beijing 100190, China*
[7]*Songshan Lake Materials Laboratory, Dongguan 523808, Guangdong, China*

SUPPLEMENTARY NOTE 1: DEVICE PARAMETERS

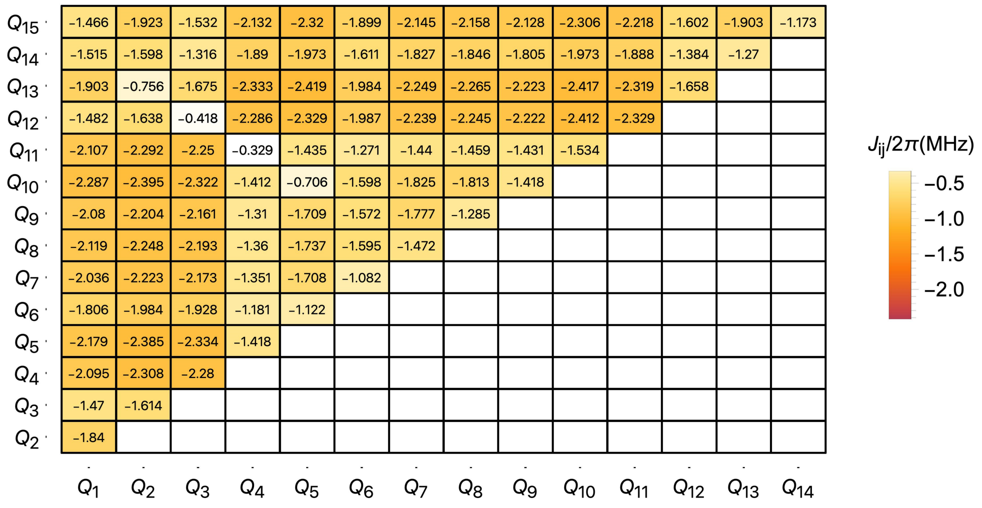

Supplementary Fig. 1. Coupling matrix between any two qubits from Q_1 to Q_{15} when $\Delta \approx -360$MHz. The coupling strength J_{ij} between qubit Q_i and Q_j is derived from energy swapping process, where two qubits are tuned at the same frequency.

In Supplementary Tab. 1, we list the main characteristics of our device with 15 out of 20 qubits. The coupling matrix between any two qubits used in the experiments is shown in Supplementary

* kaixu@iphy.ac.cn
† dzheng@iphy.ac.cn
‡ hfan@iphy.ac.cn

Fig. 1, where we relabel all qubits from Q_1 to Q_{15} for convenience. The interactions between qubits are turned off during the single-qubit gates to minimize the crosstalks between different qubit pairs. For any two qubits staying at their respective idle points, we perform two Ramsey experiments on one qubit, where the other qubit is in the ground state and the first excited state. The effect of crosstalks can be characterized by the Ramsey frequency difference δf. We have checked that all $|\delta f| \leq 0.1\,\mathrm{MHz}$. In addition, the high state fidelities $F_{\mathsf{X}_{\mathrm{all},j}}$ and $F_{\mathsf{X/2}_{\mathrm{all},j}}$ also indicate that the interactions at idle points are deeply suppressed.

	$\omega_j/2\pi$ (GHz)	$T_{1,j}$ (μs)	$T^*_{2,j}$ (μs)	$g_j/2\pi$ (MHz)	$\omega^r_j/2\pi$ (GHz)	$\omega^m_j/2\pi$ (GHz)	$F_{0,j}$	$F_{1,j}$	$F_{\mathsf{X}_{\mathrm{single}},j}$	$F_{\mathsf{X}_{\mathrm{all}},j}$	$F_{\mathsf{X/2}_{\mathrm{single}},j}$	$F_{\mathsf{X/2}_{\mathrm{all}},j}$
Q_1	4.305	~ 29	0.63	27.6	6.769	4.765	0.970	0.928	99.99%	99.24%	99.98%	99.98%
Q_2	4.530	~ 26	0.63	29.1	6.709	4.220	0.976	0.927	99.71%	99.64%	99.95%	99.98%
Q_3	4.385	~ 25	0.69	26.5	6.650	4.388	0.971	0.921	99.97%	99.88%	99.99%	99.94%
Q_4	4.433	~ 23	0.76	29.2	6.613	4.322	0.975	0.927	99.97%	99.89%	99.86%	99.78%
Q_5	4.815	~ 25	1.22	30.1	6.559	5.200	0.990	0.919	99.66%	99.04%	99.67%	99.24%
Q_6	4.090	~ 22	0.59	24.1	6.552	4.420	0.972	0.885	99.54%	99.14%	99.95%	99.77%
Q_7	4.225	~ 25	0.66	27.7	6.514	4.905	0.986	0.922	99.70%	98.40%	99.93%	99.70%
Q_8	4.855	~ 22	1.06	27.3	6.526	4.865	0.988	0.933	99.79%	98.38%	99.99%	99.11%
Q_9	4.355	~ 41	0.64	26.9	6.551	4.180	0.981	0.895	99.91%	99.45%	99.99%	99.66%
Q_{10}	4.492	~ 22	0.76	29.1	6.569	4.585	0.963	0.904	99.59%	99.69%	99.82%	98.88%
Q_{11}	5.200	~ 20	1.39	26.3	6.642	4.705	0.980	0.949	99.93%	99.70%	99.78%	99.98%
Q_{12}	4.989	~ 28	1.25	26.5	6.660	4.252	0.970	0.932	99.82%	98.84%	99.96%	99.76%
Q_{13}	5.135	~ 46	1.35	29.0	6.714	5.355	0.986	0.918	99.87%	99.32%	99.96%	99.78%
Q_{14}	5.272	~ 20	1.58	24.6	6.789	5.275	0.991	0.936	99.94%	99.65%	99.96%	99.75%
Q_{15}	4.921	~ 23	0.85	27.5	6.760	5.060	0.976	0.881	99.89%	99.14%	99.92%	99.20%

Supplementary Tab. 1. Qubit Performance. $\omega_j/2\pi$ is the idle frequency of Q_j, where single-qubit gates are applied. $T_{1,j}$ and $T_{2,j}$ are the energy relaxation time and Ramsey dephasing time of Q_j, respectively.$g_j/2\pi$ is the coupling strength between Q_j and R. $\omega^r_j/2\pi$ is the resonant frequency of readout resonator attached to Q_j. $\omega^m_j/2\pi$ is the frequency of Q_j during the measurement process. $F_{0,j}$ ($F_{1,j}$) is the measurement probability of Q_j in state $|0\rangle$ ($|1\rangle$) when Q_j is prepared in the state $|0\rangle$ ($|1\rangle$). $F_{\mathsf{X}_{\mathrm{single}},j}$ ($F_{\mathsf{X/2}_{\mathrm{single}},j}$) is the state fidelity of Q_j where we only apply one X ($\mathsf{X/2}$) gate to qubit Q_j. $F_{\mathsf{X}_{\mathrm{all}},j}$ ($F_{\mathsf{X/2}_{\mathrm{all}},j}$) is the state fidelity of Q_j where we apply X ($\mathsf{X/2}$) gates to all qubits.

SUPPLEMENTARY NOTE 2: CALIBRATION OF Z PULSE AMPLITUDE AT THE INTERACTING POINT

To realize a fully connected U gate acting on N qubits defined in Equation (7) of the main text, we bias each qubit from its idle point ω_j to the interacting point ω_I by applying a rectangular Z pulse. For Q_j initialized at $|0\rangle$, we drive with a flat-top enveloped microwave pulse at frequency ω_I and sweep Z pulse amplitude. The sequence is illustrated in Supplementary Fig. 2a where we bias other qubits in the vicinity (about 50-100 MHz) of the interacting point, taking Q_{20} as an example. The frequency arrangement of other qubits is determined by having an equal net Z-crosstalk estimated by Z-crosstalk matrix on Q_{20} compared to the case when all qubits are at the interacting point. Supplementary Figure 2b shows the probability of $|1\rangle$ of Q_{20} as a function of time t and Z pulse amplitude. For each Z pulse amplitude, the oscillation period of the probability of $|1\rangle$ is proportional to $1/\sqrt{\Delta^2+\Omega^2}$ where Δ is the frequency detuning and Ω is the driving amplitude. Therefore, the optimal Z pulse amplitude z_1 corresponds to the slowest oscillation of probability over time. We also consider another frequency arrangement of qubits which is symmetric about the interacting point to the former one and find another optimal Z pulse amplitude z_2. Then we regard the average of z_1 and z_2 as the final Z pulse amplitude.

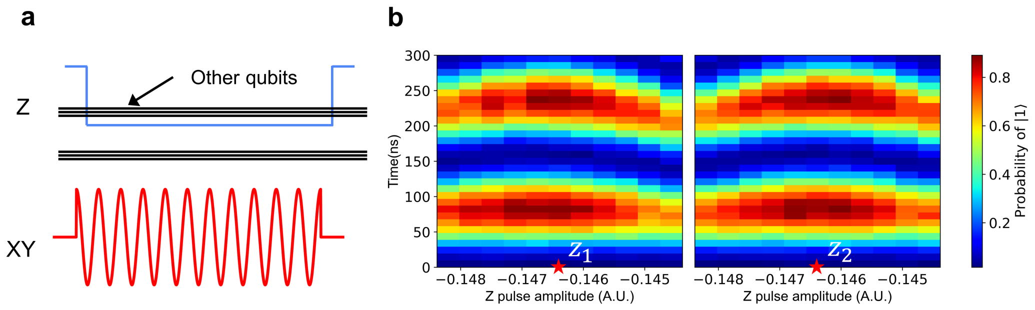

Supplementary Fig. 2. Pulse sequences and measurement results of calibration of Z pulse amplitude at the interacting point. (a) Experimental pulse sequences of calibration for Q_{20}. (b) The probability of state $|1\rangle$ as a function of Z pulse amplitude and time for two frequency arrangements.

SUPPLEMENTARY NOTE 3: NUMERICAL SIMULATION OF NEGATIVITY SPECTRA SAMPLED FROM CIRCUITS

We simulate the distribution of the negativity spectrum sampled from pseudo-random circuits by our processor without decoherence errors. The circuits include six system qubits and 3 or 9 environment qubits. System qubits are divided into two parts with 2 or 4 qubits, respectively. To demonstrate the capability of generating pseudo-randomness in our processor, we consider different circuits with layers from 1 to 8 and draw 20 instances for each circuit. The negativity spectra between subsystems are illustrated in Supplementary Fig. 3 and Supplementary Fig. 4, where we also show the distribution of the negativity spectrum of random mixed states sampled from Haar measure. Moreover, we use Kullback-Leibler divergence D_{KL} introduced in the main

text to quantify the distance between the distributions sampled from pseudo-random circuits and Haar measure. As shown in Supplementary Fig. 3, two distributions are very close from $d = 4$ to 8 when $N_B = 3$, which is verified by the histograms and D_{KL}. Thus we choose four-layer circuits to generate pseudo-random states when $N_B = 0, 1, 2,$ or 3. Similarly, we choose five-layer circuits to generate pseudo-random states when $N_B = 13, 14,$ or 15, according to Supplementary Fig. 4. The least layers of circuits to produce the negativity spectrum of random mixed states change little when the number of total qubits increases from 9 to 15, which is attributed to the full connectivity of our processor.

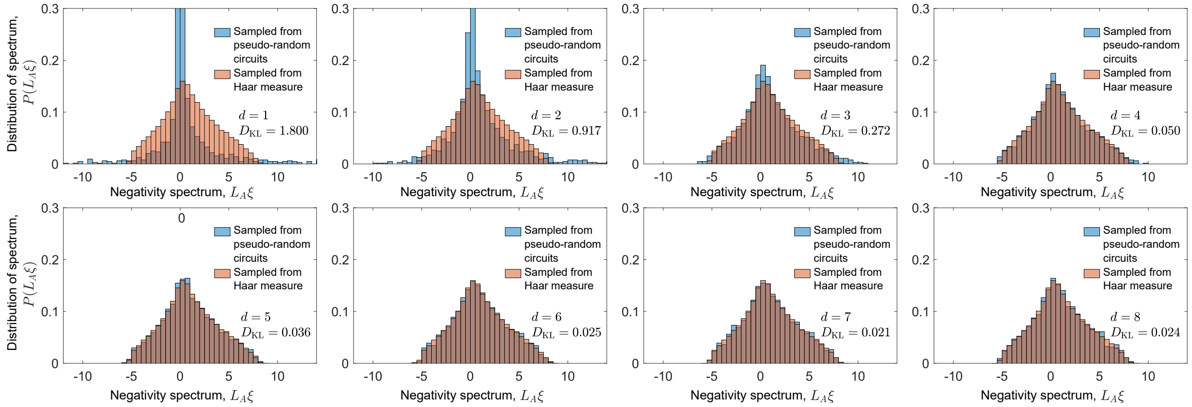

Supplementary Fig. 3. Negativity spectrum of 6 system qubits with 3 environment qubits.

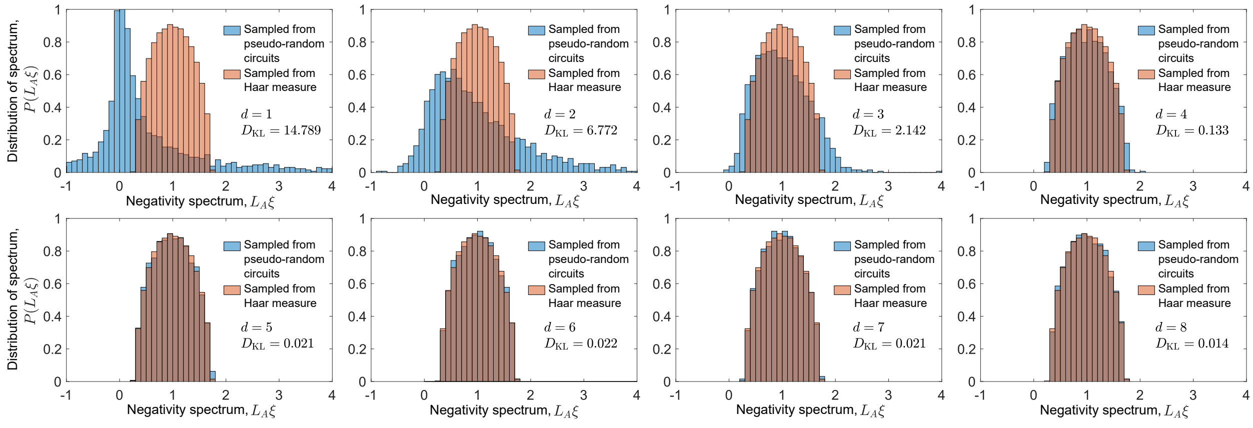

Supplementary Fig. 4. Negativity spectrum of 6 system qubits with 9 environment qubits.

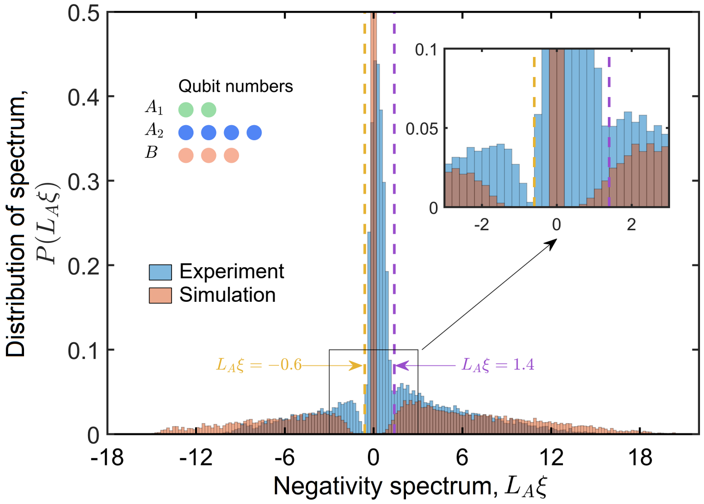

Supplementary Fig. 5. The complete negativity spectra for simulation and experiment corresponding to the Fig. 2i of the main text. Orange bars are simulation data and blue bars are experiment data. We zoom in the part of distributions near the zero in the northeast corner of figure.

SUPPLEMENTARY NOTE 4: COMPLETE NEGATIVITY SPECTRA FOR $N_B = 1$

As shown in Supplementary Fig. 5, a great number of zero eigenvalues appear in the simulation, which is predicted as a delta function in theory [1]. We can observe a similar distribution of negativity spectra in the experiment, which also includes a great number of eigenvalues in the neighborhood of zero. The distribution of these eigenvalues is confined to lie between $L_A\xi = -0.6$ and $L_A\xi = 1.4$. The remaining eigenvalues, except ones in the region $-0.6 \leq L_A\xi \leq 1.4$ are, shown in Fig. 2i of the main text, where zero eigenvalues of simulation data are excluded to compare.

SUPPLEMENTARY NOTE 5: QUANTUM STATE TOMOGRAPHY

Our protocol is based on the fact that any quantum state ρ of N qubits can be rewritten as a linear combination of Pauli operators [2],

$$\rho = \frac{1}{2^N} \sum_{i_1, i_2, \ldots, i_N} c_{i_1, i_2, \ldots, i_N} \sigma_1^{i_1} \sigma_2^{i_2} \cdots \sigma_N^{i_N}, \tag{1}$$

where $\sigma^0 = I$, $\sigma^1 = \sigma^x$, $\sigma^2 = \sigma^y$, and $\sigma^3 = \sigma^z$. Using the relation $\mathrm{Tr}(\sigma^i \sigma^j) = 2\delta_{ij}$, we obtain

$$c_{i_1, i_2, \ldots, i_N} = \mathrm{Tr}\left(\rho \sigma_1^{i_1} \sigma_2^{i_2} \cdots \sigma_N^{i_N}\right) = \langle \sigma_i^{i_1} \sigma_2^{i_2} \cdots \sigma_N^{i_N} \rangle, \tag{2}$$

which means that the combination coefficient $c_{i_1, i_2, \ldots, i_N}$ is the expect value of Pauli operator $\sigma_1^{i_1} \sigma_2^{i_2} \cdots \sigma_N^{i_N}$ in the state ρ. Define $\sigma^z \equiv |0\rangle\langle 0| - |1\rangle\langle 1|$ and $I \equiv |0\rangle\langle 0| + |1\rangle\langle 1|$. Then

$\langle\sigma^z\rangle = P(0) - P(1)$ where $P(0)$ and $P(1)$ are measurement probabilities of $|0\rangle$ and $|1\rangle$, respectively. The measurement of σ^x and σ^y can be reduced to the measurement of σ^z and I since

$$-\mathrm{Tr}(\rho\sigma^x) = \mathrm{Tr}(\rho \mathsf{Y/2}^\dagger \sigma^z \mathsf{Y/2}) = \mathrm{Tr}(\mathsf{Y/2}\rho\mathsf{Y/2}^\dagger\sigma^z), \tag{3}$$

$$\mathrm{Tr}(\rho\sigma^y) = \mathrm{Tr}(\rho \mathsf{X/2}^\dagger \sigma^z \mathsf{X/2}) = \mathrm{Tr}(\mathsf{X/2}\rho\mathsf{X/2}^\dagger\sigma^z). \tag{4}$$

Therefore, the measurement of Pauli operators is reduced to apply X/2, Y/2, and I gates to all qubits and readout simultaneously. The total time cost for a six-qubit QST is about five hours, which also includes the calibration time for the basic experiment parameters including Z pulse amplitudes of all qubits before running a QST pulse sequence. Then we can obtain a state $\tilde{\rho}$ by substituting the expected values of Pauli operators into Supplementary Equation (1). Thanks to the form of Supplementary Equation (1), $\tilde{\rho}$ has satisfied the requirements that $\mathrm{Tr}(\tilde{\rho}) = 1$ and $\tilde{\rho}^\dagger = \tilde{\rho}$. To obtain a physical density matrix, we search for a positive matrix ρ^* which minimizes the norm of $\|\tilde{\rho}-\rho^*\|_2$ in all positive matrices and regard ρ^* as the estimated density matrix of real density matrix ρ_{exp}. Supplementary Figure 6 shows the absolute values of density matrix elements of six system qubits indexed from 1 to 20, which are obtained from different pseudo-random circuits when the number of environment qubits N_B decreases from 9 to 7. The differences between density matrices for the same environment qubits are slight. Supplementary Figure 7 shows the density matrices of system qubits for $N_B = 3, 2$ and 1. The differences between density matrices are apparent because of the pseudo-randomness of the circuits. The absolute values of non-diagonal elements of density matrices increase as N_B decreases from 3 to 1, which are small compared with diagonal elements when N_B is greater than 13.

SUPPLEMENTARY NOTE 6: MODIFIED DENSITY MATRIX

Due to the decoherence errors, the purity of ρ^* obtained from quantum state tomography is less than that of ideal state ρ_U obtained from circuits without noises, i.e., $\mathrm{Tr}(\rho^{*2}) < \mathrm{Tr}(\rho_U^2)$. The average purity of 50 sampled random mixed states is given in Supplementary Tab. 2. Inspired by that, we reconstruct a density matrix ρ_{rec} after the following steps: First, we diagonalize the density matrix ρ^*, i.e., $\rho^* = \sum_i v_i|v_i\rangle\langle v_i|$, where v_i is the i-th eigenvalue and $|v_i\rangle$ is the corresponding eigenvector. Eigenvalues are sorted in decreasing order. Second, let $\rho^i_{\mathrm{rec}} = \sum_{j<i} v_j|v_j\rangle\langle v_j|$ and calculate the purity p_i of ρ^i_{rec} after normalizing ρ^i_{rec}. Third, we pick up the p_i^* which is closest to the $\mathrm{Tr}(\rho_U^2)$ and set $\rho_{\mathrm{rec}} = \rho^i_{\mathrm{rec}}$.

N_B	1	2	3	7	8	9
Average purity	0.5166 ± 0.0132	0.2712 ± 0.0126	0.1482 ± 0.0059	0.0239 ± 0.0003	0.0198 ± 0.0002	0.0178 ± 0.0001

Supplementary Tab. 2. Average purity of 50 random mixed states for N_B = 1, 2, 3, 7, 8, and 9.

The method to reconstruct a density matrix is reasonable when ρ_U is a pure state. It means that the optimal choice to estimate ρ_U is the eigenvector of ρ^* with the largest probability. We also testify the method by applying it to the other density matrices in the experiments. As shown in Fig. 3 of the main text, the negativities of ρ_{rec} are closer to the ideal ones than those of ρ^*.

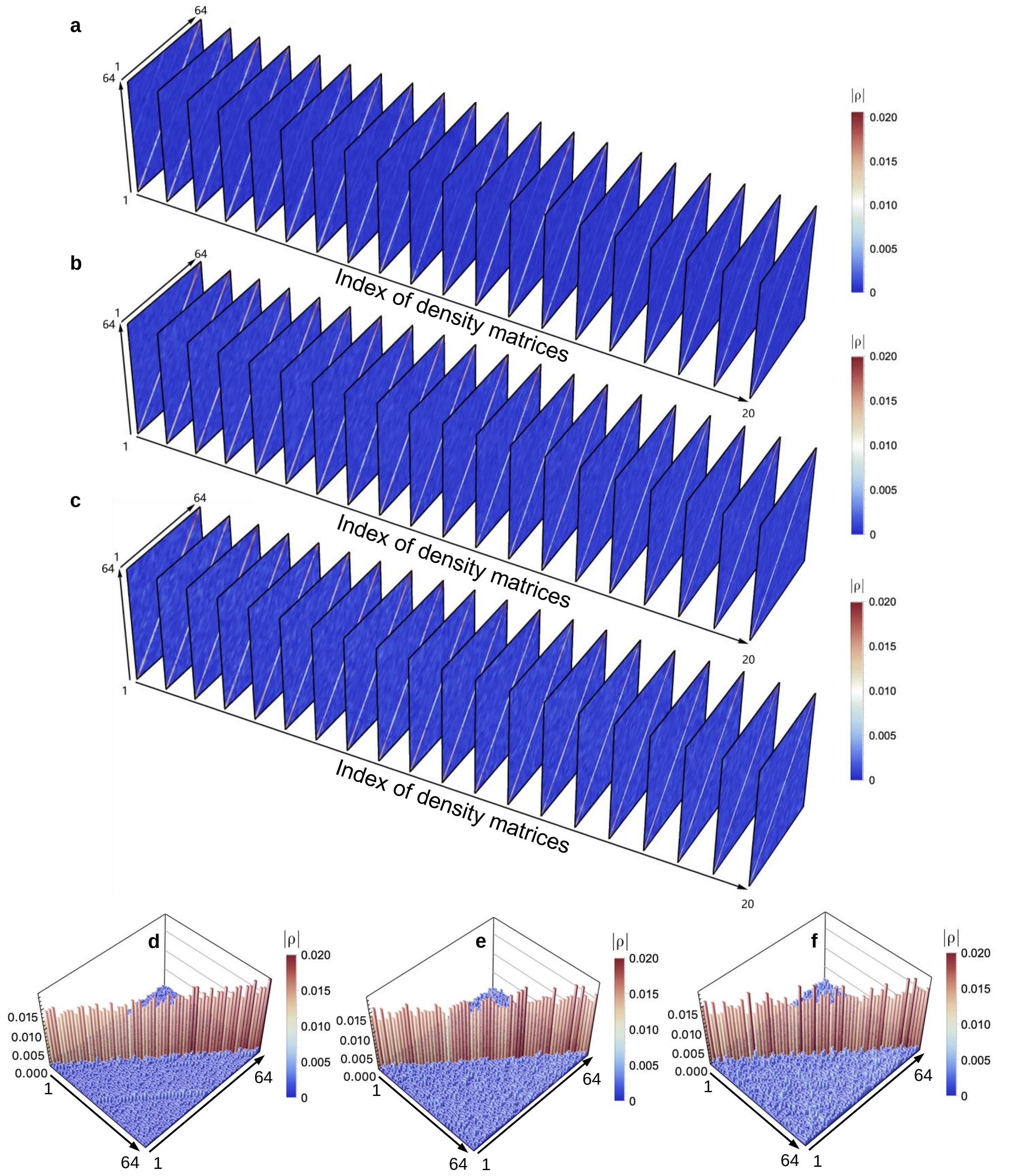

Supplementary Fig. 6. Density matrices. (a)-(c) Density matrices of 6 system qubits sampled from 20 pseudo-random circuits corresponds to $N_B = 9, 8$ and 7, respectively. (d-f) One of the density matrices shown in (a)-(c) for $N_B = 9, 8$ and 7.

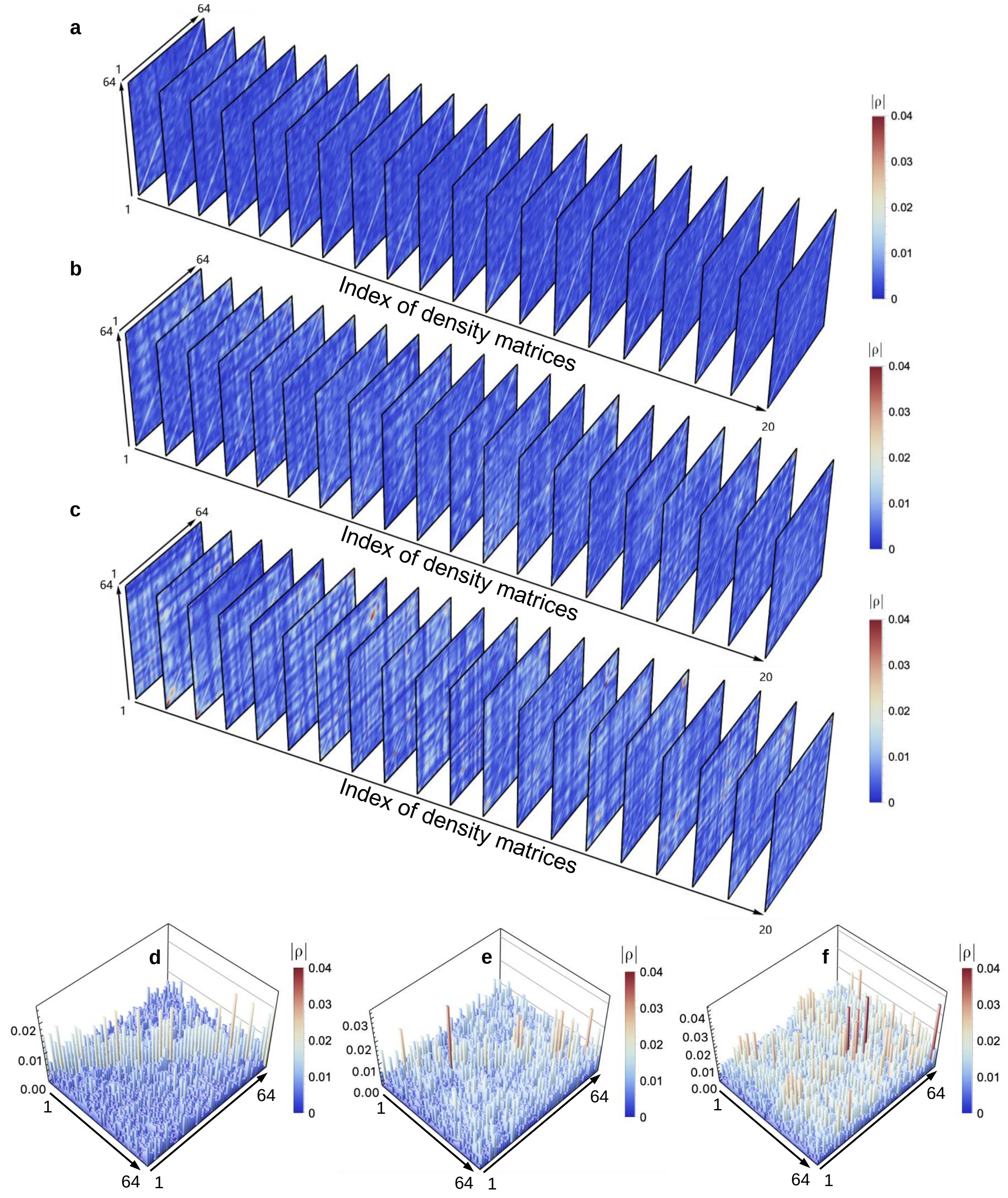

Supplementary Fig. 7. Density matrices. (a)-(c) Density matrices of 6 system qubits sampled from 20 pseudo-random circuits corresponds to $N_B = 3, 2$ and 1, respectively. (d)-(f) One of the density matrices shown in (a)-(c) for $N_B = 3, 2$ and 1.

SUPPLEMENTARY NOTE 7: IMPACT OF VARIATIONS OF COUPLING STRENGTHS BETWEEN QUBITS

We found that the variations of qubit-qubit coupling strengths do not impact the results. We substitute the free evolution operator generated by the non-uniform coupling strengths from experiments into the nine-qubit pseudo-random circuits and consider all possible choices of system qubits with $C_9^6 = 84$ cases. In Supplementary Fig. 8a, we show the distribution of negativity spectra sampled from 20 instances where we choose ten groups of system qubits shown in different histograms. They are all close to the ideal distribution sampled from random mixed states. In Supplementary Fig. 8b, we plot the average logarithmic negativities derived from the negativity spectra for 84 groups of system qubits, which are also very close to the ideal result. It means that the choice of system qubits does not impact the measurement outcome.

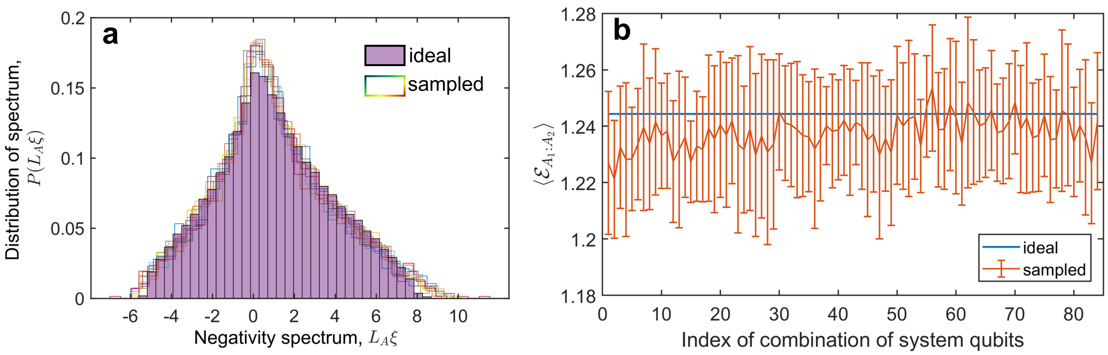

Supplementary Fig. 8. Negativity spectra and logarithmic negativities sampled from pseudo-random circuits for different groups of system qubits when $N = 9$. (a) Histograms for 10 choices of system qubits and ideal result. (b) Average logarithmic negativities for all choices of system qubits. The error bars represent standard error of the mean over circuit instances.

N	System qubits					
15	Q_3	Q_5	Q_{10}	Q_{12}	Q_{15}	Q_{19}
14	Q_1	Q_3	Q_6	Q_{12}	Q_{16}	Q_{19}
13	Q_1	Q_3	Q_9	Q_{12}	Q_{16}	Q_{18}
9	Q_1	Q_5	Q_8	Q_{10}	Q_{15}	Q_{18}
8	Q_1	Q_3	Q_5	Q_{12}	Q_{15}	Q_{18}
7	Q_1	Q_5	Q_8	Q_{10}	Q_{15}	Q_{18}

Supplementary Tab. 3. System qubits for different total qubit numbers N.

In our experiments, we also choose the following groups of qubits as system qubits when the total number of qubits N decreases from 15 to 7 as shown in Supplementary Tab. 3, which might be a sanity check to support that variations in coupling strengths do not impact the measurement outcome.

SUPPLEMENTARY NOTE 8: EFFECT OF THE SECOND EXCITED LEVELS OF TRANSMON QUBITS

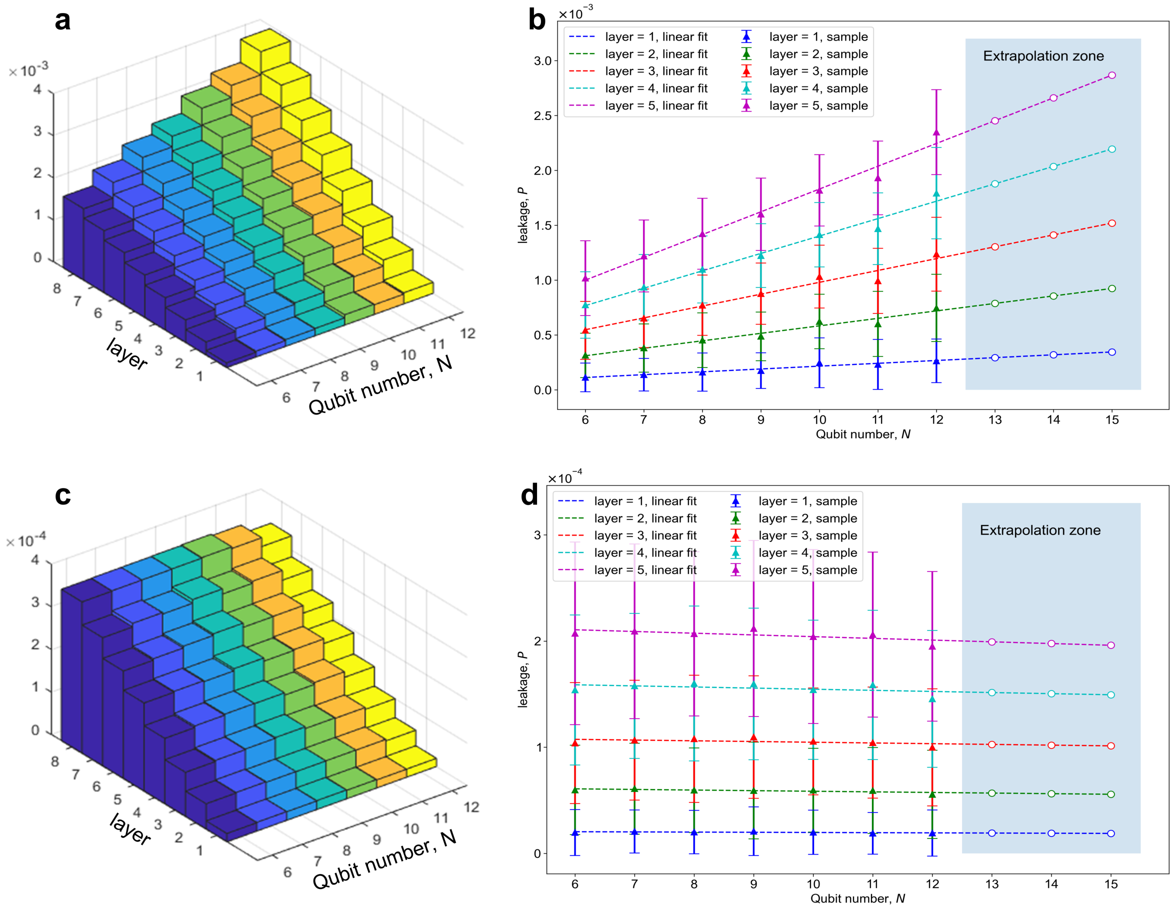

Supplementary Fig. 9. The leakage over the layer sampled from pseudo-random circuits. We sample 200 instances for $6 \leq N \leq 8$ and 50 instances for $9 \leq N \leq 12$. The leakages are fitted by linear functions. The free evolution in (a) and (b) is governed by a fully connected Bose-Hubbard model while in (c) and (d) is governed by a one dimensional Bose-Hubbard with a periodic boundary condition. The error bars in (b) and (d) represent standard error of mean over circuit instances.

The anharmonicities of our qubits η_j all lie within the range $-260\,\mathrm{MHz} < \eta_j/2\pi < -240\,\mathrm{MHz}$. In experiments, all single-qubit pulses are modulated by DRAG to remove most leakage during single-qubit gates [3], verified by the high-fidelities of single-qubit gates. Since all coupling strengths J_{ij} between qubits are far less than η_j, i.e., $J_{ij}/|\eta_j| < 1/100$, we think that the effect of the second excited levels during free evolution is minor. To quantitatively analyze the relevant effect, we numerically calculate the growth of leakage $P_j \equiv |\langle 2|\rho_j(l)|2\rangle|^2$ over the circuit layer by substituting the fully connected Bose-Hubbard model into the 40-ns free evolution part of random circuits, where $|2_j\rangle$ denotes the second excited state of Q_j. $\rho_j(l)$ is the reduced density matrix of the j-th qubit after a l-layer circuit. For simplicity, we set $J_{ij}/2\pi = 2\,\mathrm{MHz}$ for $i, j = 1, 2, \ldots, N$

such that $P \equiv \overline{P_j}$ for $j = 1, 2, \ldots, N$ where "—" indicates the statistical average of different circuit instances. As shown in Supplementary Fig. 9a and b, the average leakage increases over the layer. Furthermore, the average leakage also increases with the qubit number N linearly when the circuit layer is fixed, which can be explained by the fact that the leakage rate of one qubit is proportional to the number of its coupling qubits. As a comparison, we also calculate the leakage of a one-dimension Bose-Hubbard model with a periodic boundary condition as shown in Supplementary Fig. 9c and d. It is clear that the leakage still increases over the layer but is almost independent of the qubit number N, since each qubit is always coupled with two adjacent qubits.

We sample 200 circuit instances for $6 \leq N \leq 8$ and 50 instances for $9 \leq N \leq 12$ in Supplementary Fig. 9. To estimate the leakage for larger systems, we fit the leakage over the qubit number by linear functions and extrapolate it to $N = 13,\ 14,$ and 15. For the 15-qubit fully connected system, the leakage error after five layers is about 3×10^{-3} which is close to a single-qubit gate error. Therefore, we think that the effect of the second excited levels in our system is not adverse.

SUPPLEMENTARY NOTE 9: IMPACT OF DECOHERENCE ERRORS ON DISTRIBUTION OF OUTPUT BIT-STRING PROBABILITIES

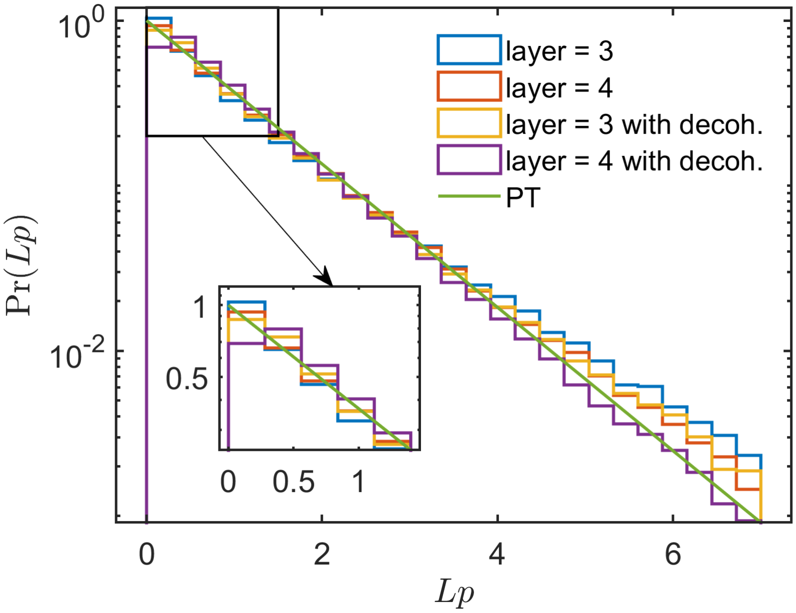

Supplementary Fig. 10. Distribution of probabilities of five-qubit bit-strings from three layer circuits, four-layer circuits, three-layer circuits with decoherence errors and four-layer circuits with decoherence errors $T_1 = 20\,\mu$s and $T_2 = 5\,\mu$s. We sample 5000 circuit instances for each histogram.

As shown in Supplementary Note 3, the negativity spectra sampled from four-layer circuits are closer to the ideal results than those of three-layer circuits when there are nine qubits in total

without decoherence errors. However, the output of three-layer circuits is closest to the PT distribution in Fig. 4 of the main text. The discrepancy might be explained by the decoherence errors in experiments. We simulate the distribution of probabilities of output bit-strings with decoherence errors by using the Lindblad master equation to capture the effect of finite energy relaxation time T_1 and Ramsey dephasing time T_2 of qubits. In Supplementary Fig. 10, the distribution sampled from three-layer circuits of five qubits with $T_1 = 20\,\mu$s and $T_2 = 5\,\mu$s is closer to the distribution sampled from four-layer circuits without decoherence errors, compared with the three-layer circuits without decoherence errors. The KL divergences $D_{\rm KL}$ between the PT distribution and the output of three-layer circuits, four-layer circuits, three-layer circuits with decoherence errors, and four-layer circuits with decoherence errors are 0.0092, 0.0017, 0.0032 and 0.0104, respectively. Therefore, if there are no decoherence errors, the output of four-layer circuits is closer to the PT distribution. The decoherence errors seem to displace the distributions such that the output of three-layer approaches to the PT distribution.

[1] H. Shapourian, S. Liu, J. Kudler-Flam, and A. Vishwanath, Entanglement negativity spectrum of random mixed states: A diagrammatic approach, PRX Quantum **2**, 030347 (2021).

[2] D. F. V. James, P. G. Kwiat, W. J. Munro, and A. G. White, Measurement of qubits, Phys. Rev. A **64**, 052312 (2001).

[3] F. Motzoi, J. M. Gambetta, P. Rebentrost, and F. K. Wilhelm, Simple pulses for elimination of leakage in weakly nonlinear qubits, Phys. Rev. Lett. **103**, 110501 (2009).
